# Expanded Hemodialysis Using a Medium Cut-Off Dialyzer for Severe Valproic Acid Poisoning: A Case Report with Real-Time Therapeutic Drug Monitoring

**DOI:** 10.3390/jcm15062220

**Published:** 2026-03-14

**Authors:** Celia Rodríguez Tudero, Avinash Chandu Nanwani, Elena Jiménez Mayor, Esperanza Moral Berrio, Marco Vaca Gallardo, Juan Daniel Díaz García, José C. De La Flor

**Affiliations:** 1 Department of Nephrology, Hospital Universitario de Salamanca, 37007 Salamanca, Spain; crodrigueztudero@usal.es; 2 Surgery Department, Faculty of Medicine, University of Salamanca, 37007 Salamanca, Spain; 3 Department of Nephrology, Hospital General de Fuerteventura, 35600 Fuerteventura, Spain; achanan@gobiernodecanarias.org; 4 Department of Nephrology, Hospital Santa Bárbara, 42005 Soria, Spain; ejimenezmay@saludcastillayleon.es; 5 Department of Nephrology, Hospital General Universitario de Ciudad Real, 13005 Ciudad Real, Spain; emoral@sescam.jccm.es; 6 Department of Nephrology, Hospital Universitario Gregorio Marañon, 28007 Madrid, Spain; marco.vaca@salud.madrid.org; 7 Department of Nephrology, Hospital General de México, Mexico City 06720, Mexico; jdiazg@clinic.cat; 8 Department of Nephrology, Hospital Central de la Defensa Gómez Ulla, 28047 Madrid, Spain; 9 Department of Medicine and Medical Specialties, Faculty of Medicine, Alcala University of Alcalá, 28805 Madrid, Spain; 10 Health Sciences Doctoral Program, Faculty of Medicine, Alcala University, 28805 Madrid, Spain

**Keywords:** valproic acid, extracorporeal toxin removal, expanded hemodialysis, medium cut-off membrane

## Abstract

**Background:** Valproic acid (VPA) poisoning has a dynamic clinical course and may require extracorporeal toxin removal (ECTR) in severe cases. Intermittent hemodialysis is the preferred ECTR technique; however, clinical experience with expanded hemodialysis (HDx) using medium cut-off (MCO) membranes in acute VPA intoxication is scarce. We describe a case of severe VPA poisoning managed with intermittent HDx and outline the clinical rationale and kinetic response. **Case Report:** A 54-year-old woman presented to the emergency department after accidental presumably ingesting approximately 4 g of VPA, with depressed consciousness (Glasgow Coma Scale 7) and metabolic acidosis (pH 7.10, HCO_3_^−^ 13 mmol/L, PCO_2_ 50 mmHg, lactate 2.8 mmol/L, ionized calcium 0.8 mmol/L, elevated anion gap). Initial plasma VPA was 262.99 µg/mL, ammonia was 14 µmol/L, and cranial computed tomography showed no acute abnormalities. ECTR was initiated in the intensive care unit as intermittent HDx using an MCO dialyzer for 4 h. Serial VPA concentrations were obtained before treatment, at 2 h, and at the end of the session to guide real-time prescription adjustment, with an increase in blood flow from 200 to 230 mL/min. **Results:** VPA decreased from 262.99 µg/mL pre-HD to 141.48 µg/mL at 2 h (46.2% reduction) and 97.81 µg/mL at 4 h (62.8% reduction), with clear improvement in the level of consciousness. A mild post-dialysis rebound was observed (100.07 µg/mL at 14 h). The patient recovered without additional ECTR and was discharged with normalized VPA levels on follow-up. **Conclusions:** In this patient, intermittent HDx with an MCO membrane was feasible, well tolerated, and associated with rapid VPA clearance and neurological recovery. Serial drug monitoring enabled bedside optimization of the dialysis prescription and post-treatment evaluation. A single HDx session was sufficient, and VPA therapy was safely reintroduced under close monitoring.

## 1. Introduction

Valproic acid (VPA) is a widely prescribed broad-spectrum antiseizure agent and mood stabilizer, characterized by a relatively narrow therapeutic index and the potential for life-threatening toxicity in overdose [1,2]. In routine clinical practice, VPA intoxication accounts for a meaningful proportion of drug-related presentations to the emergency department (ED) and intensive care unit (ICU), with a highly variable clinical phenotype shaped by the ingested dose, formulation (immediate-release [FIR] versus extended-release [FER]), co-ingestants, and interindividual susceptibility [1,2].

In valproate poisoning, severity is primarily driven by central nervous system depression, which may progress to coma and require ventilatory support, together with systemic metabolic complications such as metabolic acidosis, hyperammonemia, hepatotoxicity, and cytopenias; in the most critical presentations, cerebral edema and shock may occur [2,3,4]. VPA-associated hyperammonemia is clinically important because it can precipitate encephalopathy, even in the absence of overt hepatic failure, and has been mechanistically linked to mitochondrial dysfunction and disruption of the urea cycle [3,5].

Among the abnormalities reported in valproate overdose are hyperammonemia, elevated lactate, and hydroelectrolytic disturbances—including hypernatremia, hypocalcemia, and hypokalemia—typically in the setting of metabolic acidosis and hemodynamic compromise. These derangements are most often described in massive ingestions presenting with deep coma and hypotension that require intensive care unit admission, where multiorgan dysfunction and vasopressor support may also be necessary. In this context, extracorporeal therapies (intermittent hemodialysis and/or continuous modalities) have been used both to enhance drug elimination and to expedite correction of metabolic and electrolyte disturbances. Cytopenias, including thrombocytopenia and leukopenia, have also been reported and may complicate management in critically ill patients with valproate intoxication [6,7].

Under physiological conditions, VPA undergoes extensive hepatic metabolism, predominantly via glucuronidation and, to a lesser extent, mitochondrial β-oxidation; only a small fraction is processed through alternative oxidative pathways, and renal excretion of unchanged drug is minimal [8]. In overdose, functional carnitine depletion and saturation of primary metabolic routes may shift biotransformation toward alternative pathways, increasing the generation of potentially more toxic metabolites (e.g., ω-oxidation-derived compounds) that have been implicated in disturbed nitrogen homeostasis and impaired ammonia detoxification [5,9]. Consequently, hyperammonemia can promote astrocytic metabolic alterations (including osmotically active glutamine accumulation), neuroinflammation, and, in severe cases, an increased risk of cerebral edema [9,10].

VPA toxicokinetics are dynamic. At therapeutic concentrations, VPA is highly protein-bound; in an overdose, binding becomes saturated, increasing the unbound fraction and thereby toxicity. Together with its low molecular weight (144 Da) and low-to-moderate volume of distribution, this shift increases extracorporeal removability by hemodialysis, hemoperfusion, or hemofiltration [4,11]. After oral ingestion, peak plasma concentrations typically occur within 1–4 h, but this may be substantially delayed with FER or massive overdoses due to slowed gastric emptying and/or sustained absorption. Under therapeutic conditions, albumin binding is high (~90–95%), and the apparent volume of distribution is low-to-moderate (0.1–0.5 L/kg). In acute intoxication, the unbound fraction may rise disproportionately, contributing to non-linear toxicokinetic and increasing dialyzability. This increase in free drug, together with the relatively low volume of distribution, provides a pathophysiological rationale for extracorporeal toxin removal, as a substantial proportion of circulating VPA remains accessible to intermittent hemodialysis [4,12].

From a metabolic standpoint, VPA is eliminated primarily through hepatic biotransformation—predominantly glucuronidation and mitochondrial β-oxidation—with subsequent renal excretion of metabolites. In overdose, these pathways may become saturated, and the elimination half-life can be markedly prolonged (up to ~30 h), supporting the use of serial therapeutic drug monitoring and, in severe cases, interventions to accelerate clearance [12].

Initial management of VPA poisoning is mainly supportive, prioritizing airway protection, ventilatory and hemodynamic stabilization, and correction of metabolic derangements, with gastrointestinal decontamination when clinically appropriate [2]. However, FER and delayed absorption phenomena—including pharmacobezoar formation—require close surveillance with repeated clinical reassessment and serial concentration measurements, as peak levels may be delayed and clinically meaningful rebound can occur after apparent early improvement [2,13].

Adjunctive therapies are individualized. Levocarnitine (L-carnitine) is commonly used in selected patients, particularly in the presence of hyperammonemia and/or hepatic dysfunction, supported by mechanistic rationale and accumulating clinical experience; however, the optimal dose and route in acute poisoning remain uncertain [5]. Pharmacological strategies to lower VPA concentrations, such as carbapenems, have also been described, but require careful balancing against the risk of sustained VPA underexposure, seizure recurrence, and the need for an alternative antiseizure regimen [14].

Extracorporeal toxin removal (ECTR) is a cornerstone intervention in severe VPA poisoning [4]. Clinical reports have documented very high clearances with high-flux hemodialysis (HFHD) in massive overdoses, supporting its role in rapidly lowering plasma concentrations and facilitating neurological recovery [15]. Continuous modalities, mostly delivered as continuous renal replacement therapy (CRRT), including continuous venovenous hemodiafiltration (CVVHDF), have also been used effectively, particularly when hemodynamic instability limits the feasibility of intermittent therapies [11,16]. Activated charcoal hemoperfusion has likewise been reported, either as a standalone technique or sequentially with dialysis, and may achieve substantial VPA removal through adsorption of the circulating unbound fraction. In the EXTRIP recommendations, hemoperfusion is considered an acceptable alternative when intermittent hemodialysis is not available, supporting its role within the ECTR armamentarium for severe intoxication [4].

Although intermittent hemodialysis is generally the preferred ECTR modality in severe VPA poisoning, the published evidence largely reflects conventional high-flux prescriptions, with limited guidance on alternative extracorporeal platforms. In particular, expanded hemodialysis (HDx) using medium cut-off (MCO) membranes has not been systematically assessed in acute VPA intoxication. In this report, we describe a case of VPA poisoning treated with an HDx session and outline the clinical rationale, key implementation considerations, and the associated biochemical and neurological response.

## 2. Case Presentation

A 54-year-old female with a history of Down syndrome and Alzheimer’s disease (diagnosed in 2017) presented to the emergency department (ED) after an accidental ingestion of valproic acid (VPA). She was also diagnosed with epilepsy in 2018 following a generalized seizure. Her chronic medications included valproic acid syrup (200 mg/mL), donepezil 10 mg/day, extended-release venlafaxine 300 mg/day, clonazepam, and rosuvastatin/ezetimibe 10/10 mg/day. She had no known drug allergies.

The patient was transferred after presumably ingesting approximately 4 g of VPA (it could have been more due to the severity of the case), which corresponds to 20 mL of the 200 mg/mL syrup formulation, in a patient who was chronically taking VPA, in addition to other concomitant medications, with individual vulnerability that may also have contributed to the observed clinical phenotype. The ingestion was partially witnessed by a caregiver, who immediately alerted the emergency medical services. The patient arrived at the emergency department approximately two hours after ingestion with a progressively depressed state of consciousness, manifested by increased drowsiness and hyporeactivity.

On arrival, the Glasgow Coma Scale score was 7, consistent with severe impairment of consciousness. Examination showed mydriasis, marked drowsiness, and disorientation. Vital signs included a blood pressure of 121/61 mmHg, a heart rate of 101 beats/min, and under supplemental oxygen via a face mask (FiO_2_ 0.4, SpO_2_ 96%). Urine output during the first hours after admission was approximately 100 mL/h (≈1.4 mL/kg/h, assuming a body weight of 70 kg), consistent with preserved diuresis. A bladder catheter was placed, and urine output was monitored (with preserved diuresis during the initial evaluation).

Initial management included intravenous fluid resuscitation with 1000 mL of isotonic saline and 1000 mL of 5% dextrose, along with bladder catheterization. Given the depressed level of consciousness (GCS 7), the airway was secured by endotracheal intubation, and invasive mechanical ventilation was initiated before gastrointestinal decontamination. After airway protection, gastric lavage was performed with saline solution, followed by administration of activated charcoal. Gastrointestinal decontamination was undertaken approximately 2 h after ingestion, following an individualized risk–benefit assessment (reported large ingestion and early presentation), and the principal anticipated complication (aspiration) was mitigated by prior airway protection and close monitoring.

Laboratory testing demonstrated hemoglobin 13.9 g/dL, leukocytes 3520/mm^3^, platelets 149,000/mm^3^, glucose 68 mg/dL, and serum creatinine 1.05 mg/dL. Electrolytes and acid–base parameters were as follows: sodium 140 mmol/L, potassium 4.8 mmol/L, total calcium 8.5 mg/dL, chloride 85 mmol/L, pH 7.10, HCO_3_^−^ 13 mmol/L, PCO_2_ 50 mmHg, lactate 2.8 mmol/L, and ionized calcium 0.8 mmol/L, consistent with an elevated anion gap metabolic acidosis (GAP of 42) with a superimposed respiratory acidosis due to hypoventilation prior to airway control. After intubation, invasive mechanical ventilation was delivered in a volume-controlled mode (tidal volume 6–8 mL/kg predicted body weight, respiratory rate 18 breaths/min, PEEP 5 cmH_2_O), with FiO_2_ adjusted to maintain SpO_2_ > 94%. Before initiating HDx, the patient received intravenous hypertonic sodium bicarbonate (1 M, 100 mL ×2; total 200 mL) as metabolic support in the setting of severe high-anion gap acidosis. Liver enzymes were AST 19 U/L and ALT 46 U/L, and ammonium was 14 µmol/L, without evidence of clinically significant hepatic dysfunction. Coagulation showed no abnormalities. The plasma VPA concentration was 262.99 μg/mL, markedly above the therapeutic range. Plasma valproic acid concentrations were measured using a fluorescence polarization immunoassay. Electrocardiography showed a sinus rhythm (heart rate 90 bpm), QRS axis of −30°, narrow QRS complexes, and the QTc was at the upper limit of normal, 440 ms. Chest radiography and cranial computed tomography revealed no acute abnormalities.

Given the pronounced neurological impairment and the elevated valproic acid concentration, the patient necessitated admission to the Intensive Care Unit. HDx was subsequently initiated within the ICU environment, employing a medium cut-off (MCO) dialyzer (Nipro ELISIO™-19HX Polynephron™Osaka Japón) mounted on a Surdial™ X hemodialysis machine. This therapeutic session spanned 4 h, commencing with an initial blood flow rate of 200 mL/min, with a dialysate flow rate (Qd) of 500 mL/min and a dialysate temperature of 36 °C. The dialysate composition was as follows: sodium 140 mmol/L, potassium 3.0 mmol/L, calcium 1.5 mmol/L, magnesium 0.5 mmol/L, chloride 111 mmol/L, bicarbonate 32 mmol/L, and glucose 1 g/L. Vascular access was obtained via a temporary double-lumen 12 Fr catheter inserted into the right internal jugular vein under ultrasound guidance. Anticoagulation was achieved with systemic unfractionated heparin, administered as an initial bolus of 2000 IU followed by a continuous infusion of 500 IU/h, adjusted according to clinical monitoring, without bleeding complications. Given the absence of significant fluid overload and preserved urine output, net ultrafiltration was set at 0 mL. The prescribed dialysis dose targeted a standard 4 h session without additional convective clearance beyond the intrinsic properties of the MCO membrane. Transmembrane pressure and filtration fraction remained within recommended operating ranges throughout the session.

Therapeutic drug monitoring was performed at predefined time points: immediately before HDx, 2 h after HDx initiation, and at the end of the 4 h session. Additional post-treatment samples were obtained to evaluate post-dialysis rebound and to support real-time adjustment of dialysis parameters. The therapeutic range for valproic acid was 50–100 µg/mL.

The HDx procedure was executed utilizing an Nipro ELISIO™-19HX Polynephron™ (NIPRO CORPORATION, Osaka, Japan), representing a modality characterized by an enhanced diffusive clearance. The strategic decision to implement and subsequently optimize this extracorporeal therapy was underpinned by the specific pharmacokinetic attributes of valproic acid. These characteristics include its low molecular weight, the saturable nature of its protein binding at toxic concentrations, and a resultant increase in its free fraction, all of which render VPA particularly amenable to effective extracorporeal removal.

The ingested dose of VPA was 4 g (4,000,000 µg). Using the first measured plasma concentration (262.99 µg/mL), we calculated a very rough apparent volume of distribution as a bedside contextual estimate, acknowledging that this approach assumes complete absorption; however, in an oral overdose with sampling at ~2 h and potentially ongoing absorption, the first measured concentration is unlikely to represent the true peak exposure, making any Dose/C_measured-derived Vd highly uncertain. Thus, this calculation should not be interpreted as a true pharmacokinetic Vd. Importantly, this value should be considered only a rough apparent bedside estimate, because the ingested dose may not equal the absorbed dose, peak concentrations may occur later, and valproate toxicokinetics can be non-linear at high exposures.

Apparent Vd ≈ Dose/C_measured = 4,000,000 µg/262.99 µg/mL = 15.21 L (≈0.217 L/kg for 70 kg). Although highly uncertain, this apparent Vd falls within the range typically reported under non-toxic conditions (≈0.1–0.5 L/kg) and is provided solely for a mechanistic context to support the rationale that a substantial fraction of circulating VPA remains accessible to extracorporeal removal in the setting of saturable protein binding; it was not used to guide clinical decision-making.

The elimination rate constant (Kel) was calculated as [11]. Importantly, the Kel estimated during HDx represents the apparent elimination under extracorporeal therapy (i.e., combined extracorporeal and residual endogenous clearance), and should not be interpreted as the intrinsic pharmacokinetic elimination constant of valproic acid:

Kel (h^−1^) = (1/time) × ln (C1/C2)

and the elimination half-life (T½) as:

T½ = 0.693/Kel

Using the intermediate intra-dialysis concentration at 2 h (141.48 μg/mL), the calculated parameters were:

Kel (h^−1^) = (1/2) × ln (262.99/141.48) = 0.310 h^−1^

T½ = 0.693/0.310 = 2.24 h

Reduction (%) = [1 − (141.48/262.99)] × 100 = 46.2%

Considering the final post-dialysis concentration at 4 h (97.81 μg/mL), the estimated parameters for the entire session were:

Kel (h^−1^) = (1/4) × ln (262.99/97.81) = 0.247 h^−1^

T½ = 0.693/0.247 = 2.80 h

Reduction (%) = [1 − (97.81/262.99)] × 100 = 62.8%

All serial VPA concentrations and derived pharmacokinetic parameters are summarized in Table 1 (shown below).

Based on this intermediate determination, dialysis intensity was optimized by increasing the flow parameters, with the blood flow rate increased from 200 to 230 mL/min, to enhance extracorporeal clearance. At the end of the 4 h session, the plasma VPA concentration had decreased to 97.81 μg/mL, accompanied by a clear clinical improvement in consciousness.

After cessation of HDx, plasma VPA exhibited a mild post-dialysis rebound (97.81 to 100.07 μg/mL at 14 h). This pattern is expected in VPA poisoning and is consistent with redistribution from peripheral compartments and/or ongoing delayed gastrointestinal absorption, particularly after large ingestions. Importantly, once ECTR was no longer applied, the apparent elimination rate slowed markedly: the estimated VPA half-life increased from a few hours during HDx to 19.39 h in the 14–24 h interval.

**Figure 1:**
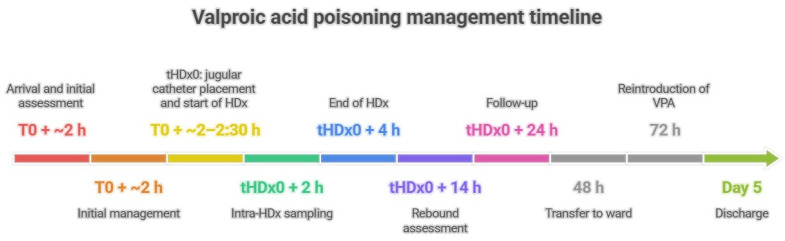
Valproic acid poisoning management timeline (CARE-compliant). Timeline of key clinical events and interventions from ingestion (T0) to discharge (day 5), including ED arrival and initial management (airway protection with endotracheal intubation followed by gastric lavage and activated charcoal), ICU admission, jugular catheter placement and initiation of expanded hemodialysis (HDx; tHDx0), intra-dialysis sampling (tHDx0 + 2 h), end of the 4 h HDx session (tHDx0 + 4 h), post-treatment rebound assessment (tHDx0 + 14 h), follow-up sampling (tHDx0 + 24 h), transfer to the ward (48 h), and reintroduction of valproic acid (VPA) at baseline regimen (72 h). Timepoints are expressed as hours from ingestion (T0) or from HDx initiation (tHDx0), consistent with the pharmacokinetic intervals reported in Table 1. Abbreviations: HDx, expanded hemodialysis; VPA, valproic acid.

After 48 h, the patient was transferred to the internal medicine ward. At 72 h, valproic acid was restarted to prevent seizure recurrence using her pre-existing maintenance regimen: valproic acid syrup (200 mg/mL) at 2–1–2 mL/day (i.e., 400–200–400 mg; total 1000 mg/day). Reintroduction was performed under close clinical monitoring and therapeutic drug monitoring with serial plasma VPA measurements. On day 5, the VPA concentration was 39.2 μg/mL, slightly below the therapeutic range, and she was discharged in a stable clinical condition. A subsequent outpatient follow-up confirmed normalization of the patient’s VPA levels without further complications. Together, these findings underscore the dynamic kinetics of VPA in overdose and the role of HDx in substantially accelerating drug clearance (Figure 1). The temporal evolution of plasma valproic acid levels and relevant laboratory parameters from emergency department arrival to day 5 is shown in Table 2.

## 3. Discussion

VPA poisoning is a common cause of drug-related toxicology presentations. Its clinical course can change over hours, depending on the ingested dose, formulation, and individual susceptibility [1,2]. This case illustrates the complexity of decision-making when serum VPA concentrations are markedly supratherapeutic but remain below the extreme levels reported in massive overdoses. It also highlights the need to integrate the overall clinical picture. In our patient, impaired consciousness was the key indication for initiating hemodialysis. Importantly, our approach was consistent with the EXTRIP principles despite the VPA levels being below the guideline concentration thresholds. We also show the feasibility of HDx by using a MCO (Nipro ELISIO™-19HX Polynephron™) dialyzer in a hemodynamically stable patient. Finally, serial therapeutic drug monitoring quantified intradialytic clearance, identified post-treatment rebound, and documented a marked prolongation of the apparent elimination phase after HD discontinuation [4].

The decision to initiate HD was driven primarily by clinical severity rather than by meeting EXTRIP’s concentration thresholds. Although the admission VPA level was markedly supratherapeutic, it did not reach the concentration cut-offs (1300 µg/mL) used by EXTRIP to support concentration-based recommended ECTR [4]. In contrast, the patient presented with profound neurological impairment (GCS 7) and severe acidemia at the EXTRIP threshold (pH 7.10; HCO_3_^−^ 13 mmol/L), both of which are explicit severity-based triggers within the EXTRIP framework [4].

The markedly low chloride at presentation (85 mmol/L) was confirmed on chart review and occurred in the context of severe high-anion gap metabolic acidosis. The rapid normalization of bicarbonate was driven by pre-dialysis administration of hypertonic sodium bicarbonate (1 M, 100 mL ×2) and bicarbonate transfer during hemodialysis (post-treatment HCO_3_^−^ 32 mmol/L).

In parallel, cerebral edema—one of the strongest EXTRIP indications—was actively excluded by brain CT, and shock was absent, thereby supporting a toxicity-driven interpretation of the depressed level of consciousness while ruling out an immediate structural explanation [4]. Taken together, our indication for HD was phenotype-based (neurological compromise) and reinforced by an objective metabolic marker (pH), consistent with EXTRIP’s emphasis on integrating clinical and biochemical severity when selecting candidates for ECTR [4].

Because the patient was hemodynamically stable, we chose an intermittent strategy to maximize instantaneous clearance and achieve a rapid reduction in circulating VPA, consistent with EXTRIP’s preference for intermittent HD as the optimal ECTR modality in this setting [4]. This decision was also pharmacokinetically coherent: in overdose, saturable protein binding increases the unbound fraction and thereby enhances extracorporeal removability, while a low-to-moderate Vd supports that a substantial proportion of the drug remains accessible to the extracorporeal circuit [4]. We implemented a HDx session using an MCO dialyzer (Elisio 19HX^TM^, Nipro Corporation, Osaka, Japan) [2,4,16]. Continuous approaches remain particularly relevant when instability prevents intermittent HD, as illustrated by the use of high-dose CVVHDF in a profoundly unstable patient with massive ingestion, where metabolic stabilization and hemodynamic improvement were prioritized under physiologic constraints [17]. In our case, although a continuous modality was considered, should instability have developed, the patient was admitted to the ICU hemodynamically stable, with preserved urine output from presentation (≈100 mL/h, subsequently increasing to ≈150 mL/h), no clinical evidence of congestion, and an overall euvolemic status.

A further strength of our management strategy was the use of serial therapeutic drug monitoring to guide real-time optimization of ECTR. Rather than relying solely on pre- and post-treatment measurements, an intermediate VPA level at 2 h quantified ongoing elimination and directly informed dialysis parameter adjustment, allowing us to intensify clearance (by increasing blood flow) based on the observed trajectory. This approach is particularly relevant in VPA poisoning, where kinetics can be non-linear due to saturable binding and where delayed absorption—especially with ER formulations or rare pharmacobezoar formation—may lead to late peaks and apparent clinical deterioration after an initial improvement [2,16]. In our patient, VPA decreased substantially during the session (262.99 mg/L pre-HD to 141.48 mg/L at 2 h and 97.81 mg/L at 4 h), supporting the capacity of intermittent HD to produce clinically meaningful reductions in plasma VPA and reinforcing its role as a cornerstone intervention when severity criteria are met [4,14]. Importantly, our stopping point was prespecified according to EXTRIP cessation guidance, which suggests discontinuing ECTR once clinical improvement is evident and/or when VPA has fallen within the therapeutic range (50–100 mg/L) [4]. Accordingly, we terminated HD at 4 h when the plasma VPA concentration reached 97.81 mg/L, in parallel with clear neurological recovery. Notably, hypocalcemia has been described in acute VPA intoxication and may reflect a multifactorial mechanism, including renal tubular dysfunction and disturbances in vitamin D metabolism. In our patient, we consider this a plausible hypothesis, but interpretation is limited because vitamin D metabolites and urinary calcium excretion were not assessed, precluding mechanistic attribution [18,19]

Post-treatment monitoring also provided important interpretive information. After cessation of HD, VPA exhibited a mild rebound (97.81 to 100.07 mg/L at 14 h), a pattern that is expected in VPA poisoning and is typically attributed to redistribution from peripheral compartments and/or ongoing delayed gastrointestinal absorption [2,4,16]. Crucially, once ECTR was discontinued, the apparent elimination rate slowed markedly, with the estimated half-life increasing to 19.39 h in the 14–24 h interval. Together, these observations highlight two practical implications emphasized in guidance and clinical experience: (i) single end-of-session concentrations may be insufficient to characterize overall exposure, and (ii) serial post-ECTR measurements are warranted to detect rebound and to support decisions regarding repeat sessions, particularly in the setting of delayed absorption risk [2,4,16,20]. In our case, the modest rebound was detected on serial measurements after the patient had already demonstrated clinical improvement, with no recurrence of symptoms; therefore, repeat HD was not required, and continued recovery supported conservative monitoring without further ECTR.

Placing our prescription in the context of the published ECTR literature highlights both what is well established and what remains less explored. Many reports of intermittent therapy in severe VPA poisoning employ high-flux membranes (often polysulfone/helixone dialyzers), and clinical improvement is commonly linked to rapid concentration decline. For example, a high-flux intermittent HD approach has been described as an “anti-coma” strategy with neurological recovery after extracorporeal removal [21], and high-flux HDF has also been reported with substantial concentration reductions during treatment [22,23]. Other authors extend the intermittent concept primarily by prolonging duration rather than changing modality: prolonged intermittent HD has been described for sustained-release cases, emphasizing that duration and repetition may be individualized in the face of delayed absorption and rebound risk [20]. In parallel, when instability predominates, continuous modalities can be selected as a pragmatic compromise, prioritizing metabolic control and hemodynamic tolerance over instantaneous clearance [17]. Case series also underscore that ECTR in VPA poisoning is often implemented as an adaptive strategy in real-world settings, with sequencing and selection influenced by concentration monitoring and bedside evolution [20]. Together, these reports support a general principle: modality and prescription are frequently dictated as much by tolerance and kinetics as by a single laboratory value.

Against this background, our case contributes a technical nuance by describing HDx with an MCO membrane in acute VPA poisoning. HDx/MCO membranes were developed primarily to broaden clearance in the middle-molecule range while maintaining albumin retention, and clinical trials and reviews have characterized the concept and its performance profile in chronic dialysis populations [24,25,26,27]. In the specific context of VPA (a small molecule whose dialyzability increases in overdose as the unbound fraction rises), the core driver of extracorporeal removal remains efficient diffusive clearance during intermittent therapy once protein binding saturates [4]. Therefore, any incremental advantage of MCO technology for VPA itself should be interpreted cautiously and not overstated; nonetheless, our observation that HDx was feasible and well tolerated, coupled with a rapid concentration decline and parallel neurological recovery, supports considering this platform as a practical option in stable patients when an MCO dialyzer is available, without implying superiority over conventional high-flux hemodialysis.

Adjunctive and alternative therapies were individualized according to biochemical phenotype and the observed response to ECTR. Levocarnitine is frequently used in selected cases, particularly when hyperammonemia and/or hepatic dysfunction are present, supported by mechanistic plausibility and accumulating clinical evidence, although optimal dosing and route remain uncertain in acute poisoning [5,9]. In our patient, their ammonia was low (14 µmol/L), and there was no clinically significant hepatic dysfunction, making omission of levocarnitine reasonable and consistent with a targeted rather than routine approach [3,5,9]. Likewise, carbapenems have been proposed as a pharmacological strategy to lower VPA concentrations; however, their use must be balanced against the risk of sustained VPA underexposure, seizure recurrence, and the need for an alternative antiseizure strategy [13]. Given the rapid biochemical response to HD and the ability to reintroduce VPA cautiously under monitoring, additional pharmacological concentration-lowering strategies were not required.

Moreover, the choice between hemodialysis and hemoperfusion (or a combined strategy) has also been discussed for valproic acid (VPA) poisoning, although the physicochemical rationale differs from other intoxications. VPA has properties that support either modality. Its low molecular weight (144 Da), moderate water solubility, and saturable protein binding at toxic concentrations favor clearance by hemodialysis. In addition, VPA is a weak acid (pKa 4.8), which facilitates adsorption onto activated charcoal at physiological pH. In vitro studies and case reports have shown efficient VPA adsorption to coated activated charcoal hemoperfusion cartridges, suggesting that hemoperfusion may also be a feasible option [28,29]. Although successful case reports have described valproic acid (VPA) poisoning treated with coated activated charcoal hemoperfusion (including one case that directly compared both techniques within the same intoxication (co-ingestion of acetaminophen and VPA), and although hemoperfusion may theoretically provide faster initial toxin removal through early cartridge adsorption, the lack of prospective comparative studies precludes any definitive conclusion regarding superiority [30].

In our case, ECTR was delivered using a MCO dialyzer, specifically the Elisio 19HX^TM^. HDx provides a broader removal spectrum than conventional high-flux hemodialysis by enhancing the clearance of larger middle molecules while maintaining a standard intermittent hemodialysis setup. Importantly, HDx can achieve a solute removal profile approaching online hemodiafiltration (OL-HDF) without increasing technical complexity or costs, as it does not require high volumes of sterile substitution fluid or specialized equipment [31,32].

From a nephrological perspective, post-dilution OL-HDF would have been an attractive option because of its well-established efficacy for middle-molecule removal and its ability to maximize convective transport. However, the patient required admission to the ICU due to severe neurological depression, and in our hospital, the ICU is not connected to the central water treatment plant. Consequently, only a portable dialysis system was available, and ultrapure water, a mandatory prerequisite for OL-HDF according to current safety and water-quality standards, could not be guaranteed at the bedside. Under these circumstances, we selected HDx with an MCO membrane, thereby overcoming the logistical and microbiological constraints that precluded OL-HDF in the critical care setting [31,32].

Nipro ELISIO™-19HX Polynephron™ is a high-efficiency synthetic dialyzer incorporating a polyethersulfone (Polynephron™) hollow-fiber membrane with an effective surface area of 1.9 m^2^, designed to provide high clearance of small and middle molecules with excellent biocompatibility [27]. The Nipro ELISIO™-19HX Polynephron™ variant has been classified as an MCO dialyzer, with an optimized pore architecture that increases permeability to larger solutes while preserving a low albumin sieving coefficient, supporting its use in intensive extracorporeal purification strategies [27]. In the setting of valproate intoxication, extracorporeal removal becomes more favorable because protein-binding saturation increases the unbound fraction, making the drug more accessible to extracorporeal clearance. In this context, the combination of a large membrane surface area, high permeability, and the ability to operate at high blood and dialysate flow rates may further enhance the elimination of free valproate and support rapid toxin reduction during HDx [33].

Evidence supporting this approach comes from comparative clinical studies. In a prospective crossover study, Maduell et al. compared conventional high-flux HD, HDx with Nipro ELISIO™-19HX Polynephron™, HDx using another MCO membrane (Theranova 400 Baxter Healthcare Corporation, Deerfield, IL, USA), and post-dilution OL-HDF. Nipro ELISIO™-19HX Polynephron™ achieved superior removal of middle and large molecules compared with standard high-flux HD, with performance approaching OL-HDF, while maintaining low albumin losses (approximately 1.5–2.5 g/session) and an excellent safety and tolerability profile [27]. Taken together, these findings support the use of the MCO dialyzer as an effective and pragmatic alternative when ultrapure water and OL-HDF are not feasible in the ICU, enabling efficient extracorporeal clearance while remaining aligned with current safety standards and real-world constraints [33].

Future studies are needed to determine whether HDx using MCO membranes offers any added benefit over conventional high-flux dialysis in acute VPA intoxication. Nevertheless, our experience indicates that this approach is feasible, well tolerated, and clinically useful as part of a severity-driven ECTR strategy in appropriately selected patients [4,31,32].

## 4. Conclusions

This case report describes the use of HDx with a MCO for acute VPA poisoning—an approach that remains less characterized than conventional high-flux intermittent HD in this clinical setting. Although the benefit of ECTR is well established when severity criteria are met, the optimal prescription (treatment duration, monitoring intensity, and membrane selection) is still largely guided by pharmacokinetic reasoning and real-time therapeutic drug monitoring rather than by standardized protocols. Our observations support a phenotype-driven approach in which profound neurological impairment and severe acidemia may justify early ECTR, even when serum VPA concentrations do not reach traditional threshold-based criteria. Moreover, these findings underscore the clinical value of intradialytic and post-ECTR sampling to quantify clearance, tailor prescription parameters, and detect rebound. Collectively, this case suggests that HDx with a MCO dialyzer is a feasible and well-tolerated option in hemodynamically stable patients in whom rapid VPA reduction and close kinetic surveillance are desirable, recognizing the inherent limitations of a single-case observation.

## Figures and Tables

**Table 1:** Plasma valproic acid concentrations and pharmacokinetic parameters during HDx.

Time Interval	C1 (µg/mL)	C2 (µg/mL)	Time (h)	Kel (h^−1^)	T½ (h)
Pre-hemodialysis to mid-hemodialysis	262.99	141.48	2	0.310	2.24
Mid-hemodialysis to post-hemodialysis	141.48	97.81	2	0.185	3.75
Post-hemodialysis to 14 h	97.81	100.07	10	−0.0023	NA
14 h to 24 h	100.07	70.00	10	0.036	19.39

Abbreviations: Kel; apparent elimination rate constant (under extracorporeal therapy when applicable); T½, elimination half-life; C, concentration; NA, not applicable/available. Negative Kel in the “post-hemodialysis to 14 h” interval reflects post-dialysis rebound (redistribution and/or ongoing absorption) rather than true elimination; therefore, T½ is not applicable, and a monoexponential model is not appropriate for this segment.

**Table 2:** Time course of key biochemical parameters.

Parameter (Units)	D 0—ED Arrival (Pre-HD)	Post-HD	24 h	48 h	D 5
Plasma VPA (µg/mL)	262.99	97.8	70.0	-	39.2
pH	7.10	7.40	7.35	7.38	7.35
HCO_3_^−^ (mmol/L)	13	36	32	28	24
Lactate (mmol/L)	2.8	1.5	1.0	1.0	0.9
Ammonium (µmol/L)	14	NA	NA	NA	NA
AST (U/L)	19	20	18	20	18
ALT (U/L)	46	46	45	40	45
Sodium (mmol/L)	140	138	137	136	138
Potassium (mmol/L)	4.8	3.8	4.1	4.0	4.1
Chloride (mmol/L)	85	100	101	103	101
Serum creatinine (mg/dL)	1.05	0.98	1.0	1.2	0.89
Total calcium (mg/dL)	8.5	8.1	8.0	8.5	8.6
Ionized calcium (mmol/L)	0.8	0.9	0.9	1.0	1.0

Abbreviations: D: day; ED: emergency department; HD: hemodialysis; VPA: valproic acid. NA: not applicable/available at that time point.

## Data Availability

No new data were created or analyzed in this study. The data used to support the findings of this study are available from the corresponding author on request (contact J.C.D.L.F., jflomer@mde.es).
